# Can Foreign Policy Make a Difference to Health?

**DOI:** 10.1371/journal.pmed.1000274

**Published:** 2010-05-11

**Authors:** Sigrun Møgedal, Benedikte Louise Alveberg

**Affiliations:** 1 Ministry of Foreign Affairs, Oslo, Norway; 2 Consultant on foreign policy and global health, Oslo, Norway

## Abstract

As part of the *PLoS Medicine* series on Global Health Diplomacy, Sigrun Møgedal and Benedikte Alveberg provide a diplomatic perspective on how foreign policy can make a difference to global health challenges.

*This article is part of the* PLoS Medicine *Global Health Diplomacy series.*

## Launch of the Foreign Policy and Global Health Initiative

In 2006 seven foreign ministers from Brazil, France, Indonesia, Norway, Senegal, South Africa, and Thailand initiated a dialogue on the inter-linkages between health and foreign policy, with a focus on how health matters to foreign policy and whether foreign policy can make a difference to health. What brought the ministers together was the realization that the state of global health has a profound impact on all nations and is deeply interconnected with trade and environment, economic growth, social development, national security, human rights, and dignity. These are challenges that go beyond the scope of ministries of health, and represent areas for which WHO (as the UN specialized agency for health) must have broader political support from member countries. Based on the ministers' analysis, the Oslo Ministerial Declaration in 2007 stated a commitment to “make impact on health a defining lens that each of the countries would use to examine key elements of foreign policy and development strategies” [1]. The ministers also decided to engage in a dialogue on how to deal with policy options from this perspective.

The need for countries to protect themselves from cross-border exposure to health risks was not a new insight in 2006. The world had already had the experience of pandemics, bioterrorism, and other threats to global health security. (The reference to security should not be understood in terms of threats to the maintenance of peace and security enshrined in the UN Charter. So far, there is no consensus on the definition of “global health security,” see Oslo Ministerial Declaration 2007 [1] and World Health Report 2007 [2]). A realization was already growing that in an interdependent world no country can manage exposure to public health risks and threats on their own, since people, animals, goods, and skills travel around the world faster than ever before in human history.

What was new was the commitment on the part of the ministers of foreign affairs to get engaged. Bringing together and building on perspectives and insights from four regions around the world, they agreed to make common vulnerability, shared risk, and shared responsibility the major starting points of their efforts. Collaboration across borders (rather than protection of “my borders”) was key to this process, as was a recognition that a nation's pursuit of pure self interest might undermine solutions that can respond to the challenges of growing interdependence. The Oslo Declaration noted that moving forward would entail a need to combine a respect for national sovereignty with the attributes of transparency, trust, accountability, and fairness.

In their follow up, the approach of the seven ministers and their teams in capitals, in Geneva, and in New York, has been practical and issue oriented, geared to capturing opportunities, engaging with each other, and seeking to communicate better and differently across traditional alliances, regions, and blocs. The agenda has now been set for health in foreign policy at both national and international levels. The process itself must now prove its value over time.

## Health Engaging Foreign Policy

While the international audience has been receptive to the “health in foreign policy” agenda, it has been harder to mainstream the awareness of the “impact on health” across the key elements of foreign policy and development strategies within the ministries of foreign affairs. Such awareness is critical for building new practices, sustaining the attention of ministers, and generating the necessary momentum for their political leadership. While the core group of countries is like-minded in terms of the purpose of their mutual engagement, they are obviously different in perceptions, priorities, and preferences, which in itself represents the very potential of such an initiative. The work of the Oslo group up to the present time shows that the health impact of foreign policy decisions needs further research. It must be better understood, assessed, and accounted for, and include the challenges of competing interests across different policy areas, within a government as well as across countries and regions.

The initiative of the seven foreign ministers has, since its inception, been in communication and collaboration with the Director-General of WHO and the Secretary-General of the UN, in order to link its work appropriately with the intergovernmental bodies, arenas, and ongoing processes in which this kind of policy dialogue may add value. WHO has in the same period strengthened its technical capacity as a hub for health-related foreign policy action, demonstrating the perceived and practical relevance of the foreign policy–global health relationship. Parallel to this development, a rapid growth of interest and networking in global health diplomacy among academic institutions and other stakeholders has taken place, harnessing the interest and endeavors of a diverse range of participants in research, development of training tools, capacity building, and support for initiatives and ongoing or upcoming negotiations. Also, these initiatives have demonstrated remarkable inclusiveness and cross-regional interest and relevance.

## The Growth of Global Health Diplomacy

Combined, these developments demonstrate that foreign policy is becoming increasingly relevant to health and that health as a shared interest can help create alliances and build bridges in international relations. As argued by Feldbaum and Michaud in their *PLoS Medicine* article “Health Diplomacy and the Enduring Relevance of Foreign Policy Interests” [3], foreign policy challenges in the health domain have increasingly moved into the “high politics” arena. Health challenges to foreign policy now cover the whole spectrum of security, economic interest, development, and dignity. This complexity of arenas and policy domains illustrates the need for transparency in dealing with the challenges of policy coherence on the one side and pragmatic issue oriented solutions on the other. A health-responsive foreign policy can succeed only if the overarching aim is to maximize the positive impact on public health and health security and this impact is monitored and brought into policy dialogue and negotiations.

## Setting the Agenda in the UN General Assembly

The close relationship between foreign policy and global health and their interdependence were recognized by all the member countries of the United Nations in the first UN General Assembly resolution on global health and foreign policy in 2008 [4]. The resolution asked the Secretary-General to recommend challenges, activities, and initiatives related to foreign policy and global health in close collaboration with the Director-General of the World Health Organization. With inputs from member country consultations, the result was a comprehensive report [5] that in many ways represents a breakthrough and makes the case for broadening the scope of foreign policy to include health. It identifies key health-related challenges that must be addressed by foreign policy makers to improve collective action to achieve global health outcomes. It also points to key foreign policy issues affecting global health and the need to improve the understanding of health implications of policies adopted in the non-health sectors. In response to the report, a second resolution [6] gave concrete and specific focus to some selected policy areas of immediate relevance to ongoing negotiations, such as pandemic influenza preparedness, access to medical products, and human resources for health.

As a topic, health in foreign policy has in this way rapidly found its place on the agenda of the General Assembly, not as an occasional, sector-specific item, but as one of the pressing foreign policy issues of our time that calls for ongoing attention and action.

## WHO as an Arena for Foreign Policy

It is increasingly clear from the ongoing work of WHO that foreign policy processes must be made to work for overcoming structural and policy-based barriers to achieving public health outcomes and global health security. The recent WHO negotiations for a global strategy and plan of action on public health, innovation, and intellectual property (WHO Public Health, Innovation and Intellectual Property Intergovernmental Working Group, WHO-PHI-IGWG), the pandemic influenza preparedness (WHO Pandemic Influenza Preparedness Intergovernmental Meeting, WHO-PIP-IGM), and the WHO process towards a global code of practice for international recruitment of health personnel together illustrate the complexity of these barriers, as is also pointed out by David Fidler in his *PLoS Medicine* article “Negotiating Equitable Access to Influenza Vaccines: Global Health Diplomacy and the Controversies Surrounding Avian Influenza H5N1 and Pandemic Influenza H1N1” [7].

The experience with the influenza A (H1N1) pandemic has clearly exposed the limitations of ad hoc arrangements when there is an urgent need for a consolidated and global response to a globally shared threat to health security. In a presentation to the UN General Assembly [8], the core group of seven countries in the Foreign Policy and Global Health Initiative noted their readiness, together with WHO, to identify how foreign policy engagement can add critical value in working towards a permanent, fair, efficient, transparent, and more predictable global framework for pandemic preparedness. A main challenge is to leverage equitable access to essential vaccines, drugs, and supplies to developing countries at the same time as to developed countries, on the basis of public health risk. When some countries can protect their citizens and others cannot, trust and solidarity among nations are threatened and all nations are exposed to greater risks. Foreign policy is called on to create and support the right political environment for making progress. This continues to be a concrete testing case for making foreign policy responsive to public health and health security.

## Health Impact in Emergencies and Post-conflict

Preventing, dealing with, and resolving conflict are well-established parts of a security and peace agenda and central to foreign policy. In a post-conflict situation, national capacity for safeguarding life and health of individuals and communities is recognized as basic to creating stability, good governance, and protection of human rights. Resilience—the ability to cope with and re-establish access to health and social services after crises, emergencies, and conflicts—depends heavily on the pre-conflict/crisis institutional capacity. It is now widely acknowledged that health indicators, such as infant mortality, are useful proxy indicators for local and national stability. Investing in capacity for protecting health and responding to health needs can therefore be understood as an investment in stability. Tensions still exist over the best ways to protect the “humanitarian space” in conflict and post-conflict situations and approaches to the transition from a humanitarian response to development under national leadership. While each situation needs to be understood in context, these are policy areas that call for more attention by all actors, including the need for a stronger evidence base and monitoring of health impacts. The 2010 review of the UN Peace-building Commission [9] may offer a concrete opportunity to apply a “health lens” to reconstruction and peace-building efforts and highlight the need for better evidence of impact, including the use of health indicators to measure and monitor progress toward peace and stability.

## The Need for Better Information and Policy Coherence

The 2009 report on “Global health and foreign policy: strategic opportunities and challenges” by the UN Secretary-General notes an urgent need to increase both the quantity and the quality of health information available to decision makers. If the impact on health is to be used meaningfully as a defining lens to examine key elements of foreign policy, the ability to collect the information, and incentives to act on it, are essential. This year, the review of the progress of countries and the international community to achieve the Millennium Development Goals will represent a major opportunity to examine impacts on health across policy domains, including policy coherence responding to public health objectives.

Achieving better policy coherence is a challenge for all countries and essential if foreign policy shall make a difference to health. The case of Brazil on global health as soft power, presented by Lee et al. in their *PLoS Medicine* article “Brazil and the Framework Convention on Tobacco Control: Global Health Diplomacy as Soft Power” [10], illustrates the strong potential of international leadership through policy consistency throughout government, such as in negotiations on access to drugs and the framework convention on tobacco control. The next report of the Secretary-General to the General Assembly on global health and foreign policy will examine ways in which foreign and health policy coordination and coherence can be strengthened at the national, regional, and international levels to serve public health, identify institutional linkages, and make concrete recommendations.

Improved governance for health now requires review and adaptation to new realities, including for WHO itself. A recent informal consultation convened by the WHO Director-General discussed how the mandate to “act as the directing and co-ordinating authority on international health work” can be understood in the radically changed landscape in which WHO now operates [11]. While this authority is being challenged in the area of development and technical assistance, there is broad agreement about the critical role for WHO in global norms and standard setting, surveillance, and the response to epidemics and other public health emergencies. The *PLoS Medicine* article “China's Engagement with Global Health Diplomacy: Was SARS a Watershed?” by Chan et al. [12] illustrates the role WHO can and must play as a partner with governments in responding to critical national health security challenges.

## Conclusion

It is in the hands of member states to direct and enable WHO to undertake its normative and standard-setting functions effectively in facing the increasingly transnational nature of health threats, to be a trusted repository for knowledge and information, and to act as an effective convener of multiple players and stakeholders that can drive appropriate convergence, innovation, and effective decision making for health in a diverse landscape.

In support of effective health governance, better evidence and best practices are needed on how foreign policy can improve policy coordination at all levels and create an improved global policy environment for health. Foreign policy practitioners need to become more aware of positive and negative impact of policy options and decisions on health outcomes. This is how foreign policy can make a difference to health.

## References

[1] Oslo Ministerial Declaration (2007). Global health: a pressing foreign policy issue of our time.. Lancet.

[2] World Health Organization (2007). The World Health Report 2007 - A safer future: global public health security in the 21st century.

[3] Feldbaum H, Michaud J (2010). Health Diplomacy and the Enduring Relevance of Foreign Policy Interests.. PLoS Med.

[4] United Nations (2009). Resolution adopted by the United Nations General Assembly on “Global health and foreign policy” (A/Res/63/33), Sixty-third session, Agenda item 44, 27 January 2009.. http://www.un.org/Docs/journal/asp/ws.asp?m=A/Res/63/33.

[5] United Nations (2009). Global health and foreign policy: strategic opportunities and challenges Note by the Secretary-General (A/64/365), United Nations General Assembly, Sixty-fourth session, Agenda item 123 Global health and foreign policy, 23 September 2009.. http://www.un.org/Docs/journal/asp/ws.asp?m=A/64/365.

[6] United Nations (2009). Resolution adopted by the United Nations General Assembly on “Global health and foreign policy” (A/64/L.16), Sixty-fourth session, Agenda item 123 Global health and foreign policy, 4 December 2009.. http://www.un.org/Docs/journal/asp/ws.asp?m=A/64/L.16.

[7] Fidler DP (2010). Negotiating Equitable Access to Influenza Vaccines: Global Health Diplomacy and the Controversies Surrounding Avian Influenza H5N1 and Pandemic Influenza H1N1.. PLoS Med.

[8] Permanent Mission of South Africa to the United Nations (2009). Statement by Ambassador Baso Sangqu, Permanent Representative of South Africa to the UN, during the introduction and adoption of resolution on Global Health and Foreign Policy (A/Res/64/L.16), 10 December 2009, General Assembly.. http://www.southafrica-newyork.net/pmun.

[9] United Nations Peacebuilding Commission (2010). Home page.. http://www.un.org/peace/peacebuilding.

[10] Lee K, Chagas LC, Novotny TE (2010). Brazil and the Framework Convention on Tobacco Control: Global Health Diplomacy as Soft Power.. PLoS Med.

[11] WHO (2010). The future of financing for WHO. Report of an informal consultation convened by the Director-General, Geneva 12–13 Jan 2010.. http://www.who.int/dg/future_financing/who_dgo_2010_1/en/index.html.

[12] Chan L-H, Chen L, Xu J (2010). China's Engagement with Global Health Diplomacy: Was SARS a Watershed?. PLoS Med.

